# Overexpression of Salicylic Acid Carboxyl Methyltransferase (*CsSAMT1*) Enhances Tolerance to Huanglongbing Disease in Wanjincheng Orange (*Citrus sinensis* (L.) Osbeck)

**DOI:** 10.3390/ijms22062803

**Published:** 2021-03-10

**Authors:** Xiuping Zou, Ke Zhao, Yunuo Liu, Meixia Du, Lin Zheng, Shuai Wang, Lanzhen Xu, Aihong Peng, Yongrui He, Qin Long, Shanchun Chen

**Affiliations:** Citrus Research Institute, Southwest University/Chinese Academy of Agricultural Sciences, Chongqing 400716, China; 15223141445@163.com (K.Z.); lynzyl0416@163.com (Y.L.); dmx18834196621@163.com (M.D.); zlin960911@163.com (L.Z.); wangshuai05072021@163.com (S.W.); lzx@cric.cn (L.X.); pengaihong@cric.cn (A.P.); heyongrui@cric.cn (Y.H.); longqin@circ.cn (Q.L.)

**Keywords:** citrus, disease tolerance, HLB, SA, MeSA, *CsSAMT1*

## Abstract

Citrus Huanglongbing (HLB) disease or citrus greening is caused by *Candidatus* Liberibacter asiaticus (Las) and is the most devastating disease in the global citrus industry. Salicylic acid (SA) plays a central role in regulating plant defenses against pathogenic attack. SA methyltransferase (SAMT) modulates SA homeostasis by converting SA to methyl salicylate (MeSA). Here, we report on the functions of the citrus *SAMT* (*CsSAMT1*) gene from HLB-susceptible Wanjincheng orange (*Citrus sinensis* (L.) Osbeck) in plant defenses against Las infection. The *CsSAMT1* cDNA was expressed in yeast. Using in vitro enzyme assays, yeast expressing CsSAMT1 was confirmed to specifically catalyze the formation of MeSA using SA as a substrate. Transgenic Wanjincheng orange plants overexpressing *CsSAMT1* had significantly increased levels of SA and MeSA compared to wild-type controls. HLB resistance was evaluated for two years and showed that transgenic plants displayed significantly alleviated symptoms including a lack of chlorosis, low bacterial counts, reduced hyperplasia of the phloem cells, and lower levels of starch and callose compared to wild-type plants. These data confirmed that *CsSAMT1* overexpression confers an enhanced tolerance to Las in citrus fruits. RNA-seq analysis revealed that *CsSAMT1* overexpression significantly upregulated the citrus defense response by enhancing the transcription of disease resistance genes. This study provides insight for improving host resistance to HLB by manipulation of SA signaling in citrus fruits.

## 1. Introduction

Huanglongbing (HLB) disease or citrus greening is associated with non-cultured, phloem-limited *Candidatus* Liberibacter asiaticus (Las) and is the most devastating disease to the citrus industry around the world [1]. HLB disease continues to cause substantial economic losses in many affected areas of the world [2,3]. In China, around 50 million diseased trees have been destroyed in the last six years. In the past decade, the disease has had a major impact on the citrus industry in Florida resulting in the decline of production from 170 million boxes to less than 45 million boxes in 2015–2018 [4]. HLB is mainly spread by the Asian citrus psyllid (ACP), *Diaphorina citri*, or experimentally by grafting [1]. To date, there is no radical cure to eliminate HLB disease. However, many HLB management strategies have been constructed to prevent HLB spread including controlling psyllid populations, the destruction of infected trees, and the use of HLB-free plants [3].

Almost all citrus cultivars including sweet oranges, mandarin lemons, and grapefruits are susceptible to HLB disease and no resistant commercial varieties are currently available for citrus farmers [5]. Recently, several genes from citrus have shown the potential to improve plant tolerance or resistance to HLB disease [6,7,8,9]. Understanding the molecular mechanisms and the regulatory processes involved in the citrus response to HLB could stimulate renewed efforts to develop more effective and economical HLB control methods.

Accumulating evidence has shown that plant systemic acquired resistance (SAR) plays an important role in the response to HLB. Martinelli et al. [10] found that SAR response was inadequately activated by Las infection in young leaves of Valencia sweet orange (*Citrus sinensis*) and generally considered young tissues as sites where most new infections occur. It has also been suggested that Las infection represses citrus SAR defense to promote the establishment of Las colonies in the phloem [11,12]. Previously, we have shown that HLB-tolerant Sour pomelo possesses a strong SAR response to Las infection [13]. The Non-expressor of Pathogenesis-Related gene 1 (*NPR1*) is a key regulator of plant SAR [14]. The overexpression of *Arabidopsis NPR1* in sweet oranges activates the expression of transcription of genes involved in the plant SAR response and enhances resistance to HLB [15,16]. Wang et al. [17] found that the response of NPR1-like genes to Las infection in HLB-tolerant ‘Jackson’ grapefruit trees (*Citrus paradisi*) is stronger than that in HLB susceptible ‘Marsh’ grapefruit trees. Furthermore, we recently showed that the overexpression of a *NPR1*-like (*CiNPR4*) gene from HLB-tolerant ‘Jackson’ grapefruit enhanced Huanglongbing resistance in Wanjincheng orange plants [9]. Collectively, these data demonstrate that manipulating the plant SAR has potential applications in the breeding of HLB-resistant citrus.

Hormone SA is a central signal in the activation of the SAR and has multiple roles in pathogen-associated molecular pattern-triggered immunity (PTI) and effector-triggered immunity (ETI) in plants [18]. When plants are invaded by pathogens, the accumulation of SA at the infected site activates the expression of downstream defense genes to trigger local acquired resistance (LAR) against pathogenic infections. Simultaneously, the activated SA signal in the infected tissue is transported through the phloem to the systemic tissue where it activates the SAR to inhibit other pathogenic infections [18,19]. Methyl salicylate (MeSA) is a SA derivative that is a critical phloem-mobile molecule for SA-mediated SAR signaling in response to pathogenic invasion [19]. MeSA accumulates in the infected tissue and is translocated through the phloem to the distal uninfected tissue. Once it is in the distal uninfected tissue, MeSA is converted back by salicylic acid-binding protein 2 (SABP2) to bioactive SA which triggers the SAR to suppress further infection and pathogenic spread [19,20].

SA carboxyl methyltransferase (SAMT) is responsible for the formation of MeSA from SA using S-adenosyl-L-methionine (SAM) as the methyl donor [21]. In tobacco, NtSAMT1 is required for the generation of the SAR-signal at tissues infected with the tobacco mosaic virus [19]. Also, silencing the *NtSAMT1* gene has been shown to block the SAR [19]. Transgenic soybeans overexpressing *GmSAMT1* exhibit resistance to soybean cyst nematode [22,23]. *SAMT* overexpression in transgenic tomato plants results in delayed disease symptoms following *Xanthomonas campestris* pv. *Vesicatoria* infection [24]. However, in *Arabidopsis*, the overexpression of rice *OsBSAMT1* leads to reduced disease resistance in transgenic plants [25]. Interestingly, both the overexpression and inhibition of *AtSAMT1* in *Arabidopsis* reduce disease resistance in transgenic plants [26].

In our previous study, we found a citrus SAMT gene *CsSAMT1* was related to the Las-induced accumulation of MeSA and plant tolerance to HLB [13]. Thus, we hypothesize that *CsSAMT1* plays a role in citrus HLB tolerance by modulating the SA and MeSA signals. Here, we report on the detailed functions of *CsSAMT1* in the response of citrus to Las infection. The biochemical activity of the CsSAMT1 enzyme was characterized using yeast-expressed recombinant CsSAMT1. The roles of *CsSAMT1* in citrus HLB tolerance were evaluated by overexpressing *CsSAMT1* in the HLB-susceptible Wanjincheng orange. Our results showed that the overexpression of *CsSAMT1* significantly increased SA and MeSA signals, activated the transcription of defense genes, and enhanced the tolerance to Las in Wanjincheng oranges. The potential mechanisms of HLB tolerance conferred by *CsSAMT1* were revealed by surveying changes in hormone levels and by characterizing the anatomical and transcriptomic responses to HLB in citrus.

## 2. Results

### 2.1. Identification of a Citrus SAMT from WANJINCHENG Oranges

**Figure 1:**
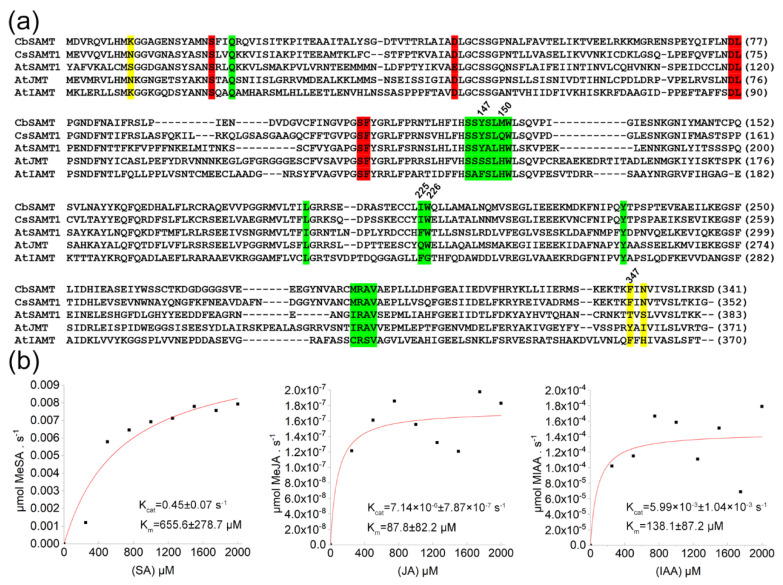
Characterization of the structural and kinetics of CsSAMT1. (**a**) Partial sequence alignment of representative Scheme 133053. AtSAMT1 is *Arabidopsis* SAMT1 (BT022049), AtJMT is *Arabidopsis* JMT (AY008435), AtIAMT is *Arabidopsis* IAMT (At5g55250), CsSAMT1 is Wanjincheng orange SAMT (*Citrus sinensis* (L.) Osbeck, Cs1g24440). (**b**) The substrate specificities of purified recombinant CsSAMT1. 0, 250, 500, 750, 1000, 1250, 1500, 1750, and 2000 µM SA, JA and IAA were used to determine the biochemical activities of CsSAMT1. After 30 min of inoculation, the MeSA, MeJA and MeIAA contents in the reaction mixture were determined using enzyme-linked immunosorbent assay (ELISA) kits for MeSA, MeJA, and MeIAA (Jiweibio, Shanghai, China). The red line represents the nonlinear least-squares fit of the initial velocities versus the SA, JA or IAA concentration to the Michaelis-Menten equation. K_cat_ was calculated by dividing the maximum velocity with the micromoles of the enzyme used. Errors indicate the standard deviations of the means of three tests. SA, salicylic acid; MeSA, methyl salicylate; JA, jasmonic acid; MeJA, methyl jasmonic acid; IAA, indole-3-acetic acid; MeIAA, methyl indole-3-acetic acid.

In our previous study, we showed that a putative citrus SAMT gene (*CsSAMT1*) positively responded to Las infection [13]. Here, a coding sequence of this gene was cloned from Wanjincheng orange. The CsSAMT1 showed the highest level of sequence homology (60.1%) to CbSAMT from Clarkia among characterized SAMT-like proteins (Figure 1a). Also, most of the amino acid residues in the active sites of CsSAMT1 were conserved compared to CbSAMT (Figure 1a). SAMTs belong to the SABATH family of methyltransferases that include jasmonic acid methyltransferase (JMT) [27] and indole-3-acetic acid methyltransferase (IAMT) [28]. The alignment of the peptides also showed that CsSAMT1 had high levels of sequence homology to AtJMT (45.6%) and AtIAMT (32.8%) and the three proteins had highly conserved active sites (Figure 1a).

The *CsSAMT1* gene was inserted into the pPIC9K plasmid and transferred into the *Pichia pastoris* strain GS115 to express the CsSAMT1 recombinant protein in yeast cells (Figure S1). Enzymatic activity analysis showed that the recombinant CsSAMT1 protein had methyltransferase activity against SA when using SAM as a methyl donor (Figure S1). To further identify the substrate specificities of the CsSAMT1 enzyme, the kinetic properties of purified CsSAMT1 recombinant protein were determined and compared to SA, jasmonic acid (JA), and indole-3-acetic acid (IAA) (Figure 1b). At 25 °C, CsSAMT1 possessed the highest Km (655.6 ± 278.7 µm) and Kcat (0.45 ± 0.07 s^−1^) values using SA as a substrate, compared to JA (Km = 87.8 ± 82.2 µm; Kcat = 7.14 × 10^−6^ ± 7.87 × 10^−7^ s^−1^) and IAA (Km = 138.1 ± 87.2 µm; Kcat = 5.99 × 10^−3^ ± 1.04 × 10^−3^ s^−1^) as substrates. The catalytic efficiency (kcat/Km) of CsSAMT1 against SA was 687.62 s^−1^.m^−1^ which was also markedly higher compared to JA (0.09 s^−1^.m^−1^) and IAA (72.31 s^−1^.m^−1^). These data confirmed that the CsSAMT1 is a salicylate acid carboxyl methyltransferase that specifically catalyzes the formation of MeSA using SA as a substrate.

### 2.2. Production of Wanjincheng Orange Transgenic Plants Overexpressing CsSAMT1

**Figure 2:**
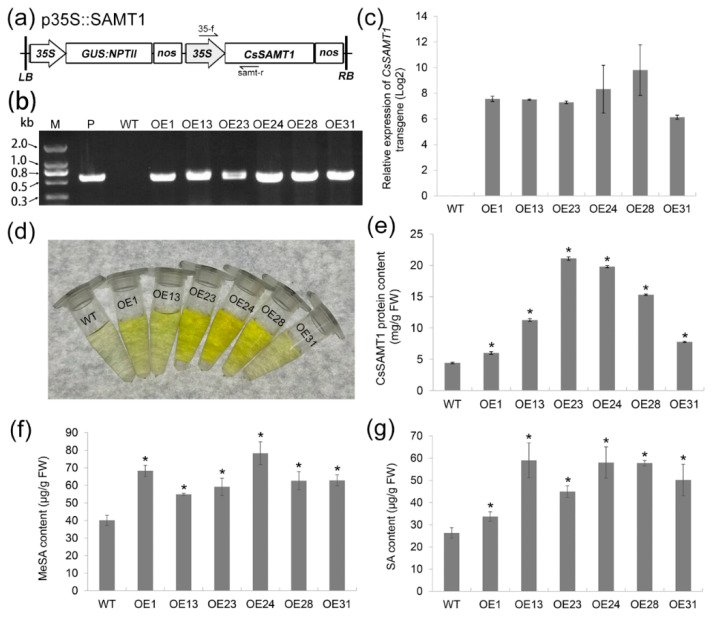
Production of transgenic Wanjincheng orange plants overexpressing *CsSAMT1*. (**a**) T-DNA structure of the p35S::SAMT1 construct used in the citrus transformation. *35S*, tobacco *Cauliflower mosaic virus 35S* promoter; *GUS:NPTII*, fusion of β-glucuronidase and neomycin phosphotransferase genes (for the screening of citrus transformants); *nos*, nos terminator; *LB*, left border; *RB*, right border. 35-f/samt-r primers for the identification of transgenic plants by PCR. (**b**) PCR confirmation of transgenic plants. Using 35-f/samt-r primers shown in (**a**), transgenic plants were confirmed by PCR. (**c**) Relative expression of *CsSAMT1* transgene in transgenic plants. The citrus *actin* (XM_006464503.3) was used as the reference gene for transcript normalization. Compared to Wild type (WT) plants, the relative expression of *CsSAMT1* in transgenic plants was determined using qRT-PCR. Log2 values of relative expression were presented here. (**d**,**e**) ELISA assay of SAMT protein levels in transgenic plants. Protein levels were calculated based on the weight of fresh leaves. (**f**,**g**) Determination of MeSA and SA contents in transgenic plants. SA and MeSA were extracted from fully matured leaves and were determined using the plant SA and MeSA ELISA kits. Vertical bars indicate the standard deviations of the means of six tests. * represent significant differences from WT controls based on a Tukey’s test (*p* < 0.05). M, DNA marker; P, p35S::SAMT1 plasmid; WT, wild type; OE#, transgenic plants; SA, salicylic acid; MeSA, methyl salicylate.

To understand the functions of *CsSAMT1* in citrus responses to HLB, the p35S::SAMT1 vector containing the *CsSAMT1* gene under the control of a strong promoter 35S (Figure 2a) was introduced into Wanjincheng oranges by *Agrobacterium*-mediated epicotyl transformation. The transgenic plants were identified by PCR analysis (Figure 2b). The expression levels of *CsSAMT1* in the transgenic plants were evaluated by qRT-PCR. The transformation obtained six independent lines (OE1, OE13, OE23, OE24, OE28 and OE31) showed significant overexpression of *CsSAMT1* transcripts (Figure 2c and Figure S2). Enzyme-linked immunosorbent assay (ELISA) results showed that the CsSAMT1 protein levels in the transgenic lines increased between 1.4 and 4.8 folds compared to the wild type (WT) plants (Figure 2d,e). After the transgenic lines were planted in a greenhouse, their phenotypes showed no obvious differences compared with the WT plants during two years of observation (data not shown).

The effect of *CsSAMT1* overexpression on MeSA and SA accumulation in transgenic plants was determined by ELISA (Figure 2f,g). All the transgenic plants showed increased MeSA levels that were significantly higher than in WT plants. SA levels in the transgenic lines were also significantly increased compared to WT plants. These results showed that the overexpression of *CsSAMT1* can simultaneously promote the accumulation of MeSA and SA in citrus.

### 2.3. Overexpressing CsSAMT1 Enhances Tolerance to HLB in Transgenic Plants

**Figure 3:**
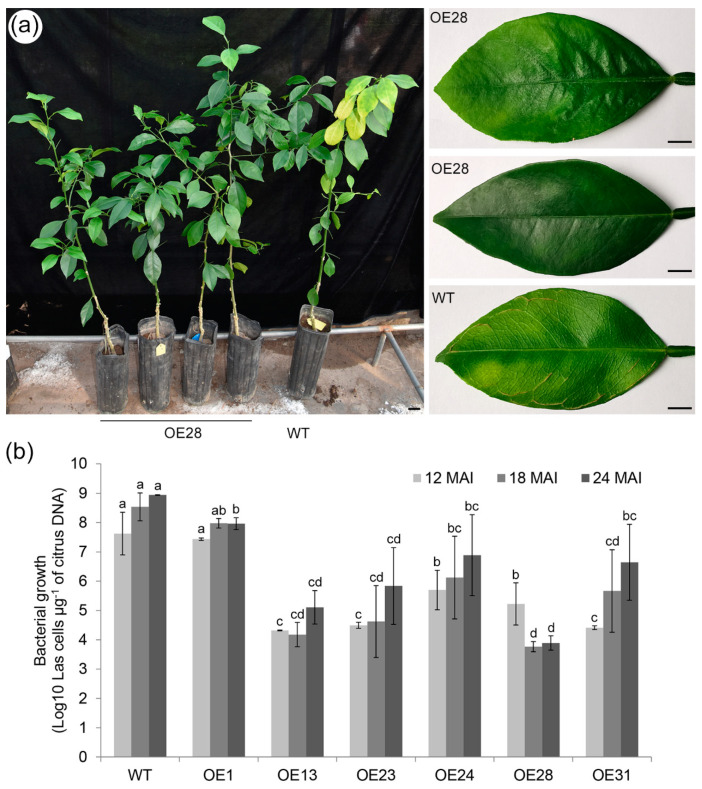
Evaluation of Citrus Huanglongbing (HLB) tolerance in transgenic citrus plants grown in a greenhouse. (**a**) HLB symptoms in the transgenic plants and a wild type (WT) control 12 months after infection (MAI). (**b**) Quantitative analysis of Las bacterial growth at 12, 18 and 24 MAI. The bacterial populations (Las cells μg^−1^ of citrus DNA) were investigated using qPCR. Standard errors were calculated from three or four plants per line. Different letters on the top of the bars indicate significant differences from the WT control based on a Tukey’s test (*p* < 0.05). WT, wild type; OE#, transgenic plants. Bar = 1 cm.

The tolerance of transgenic plants to HLB disease was determined using the grafting infection method [29]. On average, three or four plants per line were grafted with the axillary buds containing the Las pathogen. After 12 months of infection, chlorosis or mottled yellow symptoms were detected in new leaves and new flushes of the WT control plants (Figure 3a and Figure S3). Prominent veins were found in some infected WT leaves (Figure 3a). We noted no obvious symptoms in all six of the transgenic lines overexpressing *CsSAMT1* (Figure 3a and Figure S3). Moreover, during the two years of greenhouse evaluation, there were no obvious symptoms observed in the transgenic lines (Figure S4). The growth of pathogenic bacteria was determined by qPCR at 12, 18 and 24 months after Las infection. Our results showed that the Las growth in the transgenic plants (OE13, OE23, OE24, OE28, and OE31) was significantly slower than in WT plants (Figure 3b and Table S1). The OE1 line showed no significant changes in Las growth compared to WT plants (Figure 3b). The OE13 and OE28 plants had the lowest average levels of las counts. These data showed that the overexpression of *CsSAMT1* can markedly alleviate Las-induced symptoms and repress pathogenic growth in Wanjincheng oranges.

### 2.4. Anatomical Responses to Las Infection in Transgenic Plants

**Figure 4:**
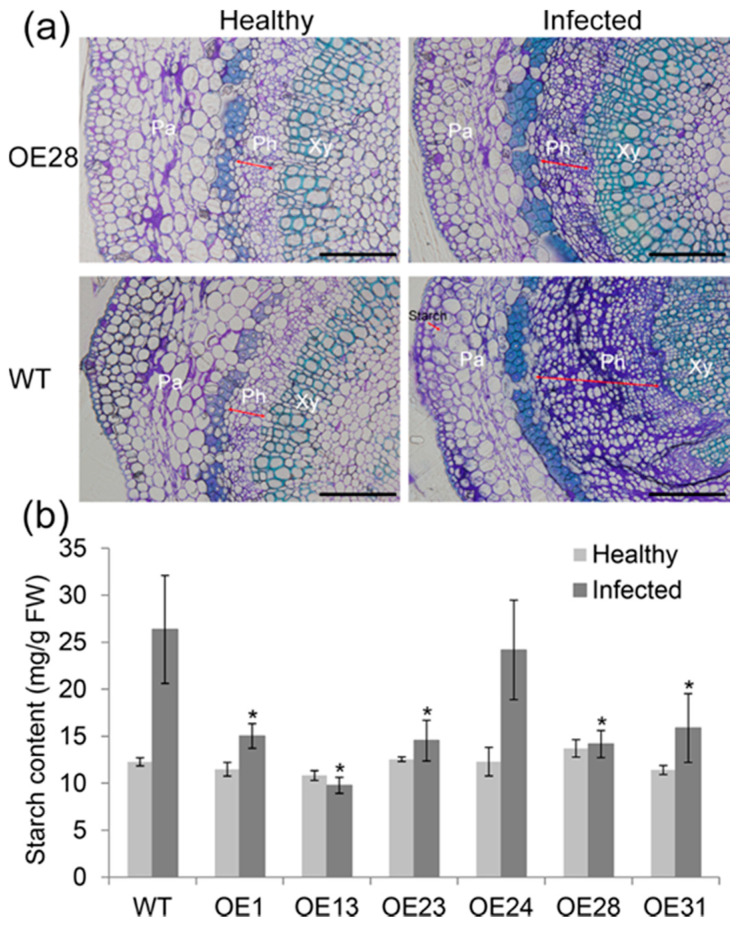
Phloem and starch changes in response to *Candidatus* Liberibacter asiaticus (Las) in the leaves of transgenic plants. (**a**) Anatomical analysis of the midrib phloem tissues of infected and control leaves 24 months after Las infection. The midribs were collected from the leaves with representative symptoms shown in Figure 3a. The slides were stained with methylene blue-azure A and basic fuchsin. Phloem over-proliferation and starch particles (arrows) were observed predominantly in the Las-infected WT leaves. (**b**) The starch contents in leaf tissues 24 months after Las infection. Starch contents are expressed relative to fresh weight. The vertical bars indicate the standard deviations of the means of three tests. * represents significant differences from the WT control based on a Tukey’s test (*p* < 0.05). WT, wild type; OE#, transgenic plants. Pa, parenchyma; Ph, phloem; Xy, xylem. Bar = 20 μm.

To compare differences in cell structure in response to Las infection between transgenic and WT plants, the anatomical changes of the midribs were analyzed using light microscopy. Significant differences were detected in the midribs between transgenic and WT control plants 24 months after Las infection (Figure 4a and Figure S5). Compared to the healthy plants, the infected plants had increased amounts of phloemic cell layers and smaller phloemic cells. We also noted that the number of cell layers in the phloem of the transgenic plants was less than in the WT controls. Basic fuchsin staining showed thickening of the cell wall in the phloem of transgenic plants that were markedly weaker than in the WT plants. The accumulation of starch gains was observed in the phloem parenchyma cells in the infected WT midribs, but this was minimally observed in the infected transgenic midribs. Starch quantification further confirmed that the starch levels in the transgenic leaves increased more slowly than the WT controls after Las infection (Figure 4b).

**Figure 5:**
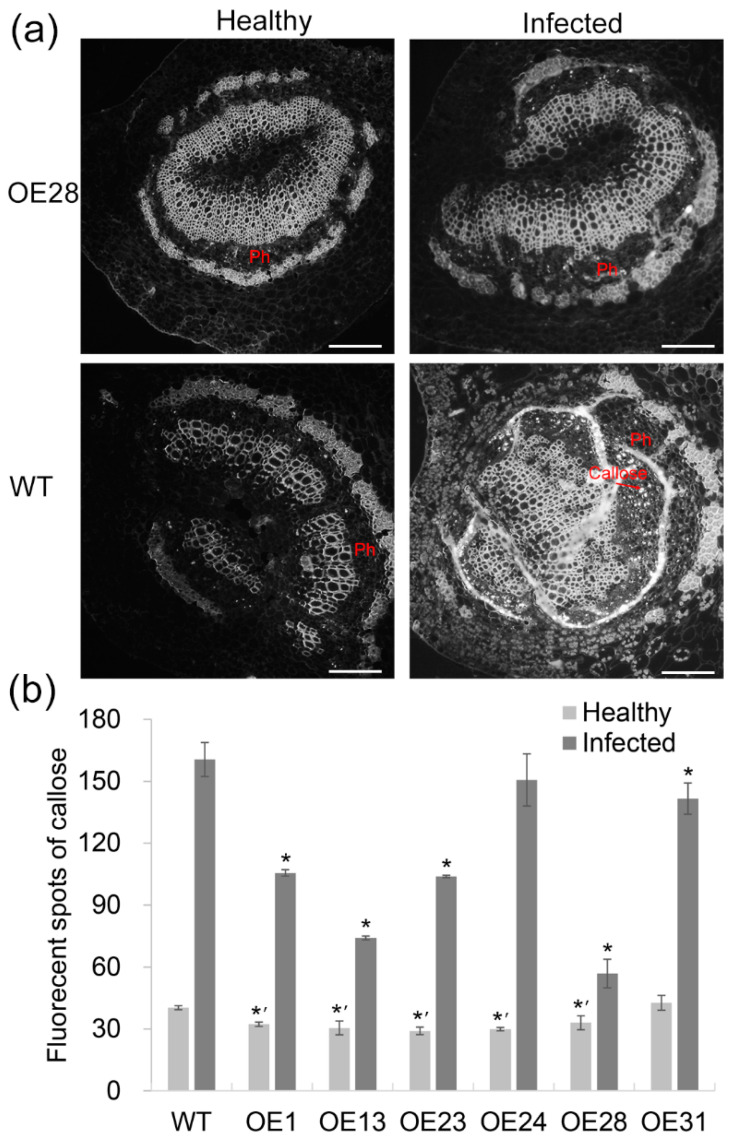
Comparison of callose deposition in the phloem of the transgenic and WT plants. (**a**) Anatomical analysis of callose in the midrib phloem tissues 24 months after *Candidatus* Liberibacter asiaticus (Las) infection. The slides were stained with 0.05% aniline blue solution and observed using a fluorescent microscope with a UV filter. Light spots represent callose deposits in the phloem. (**b**) Callose quantification in the leaf midribs 24 months after Las infection. Callose was quantified by the number of fluorescent spots in the phloem from each sample. Ten slides were counted per sample for each test. The vertical bars indicate the standard deviations of the means of three tests. * and *’ represent statistically significant differences from healthy and Las-infected WT controls, respectively, based on a Tukey’s test (*p* < 0.05). WT, wild type; OE#, transgenic plants. Ph, phloem. In (**a**), bar = 100 µm.

Callose deposition was determined by aniline blue staining. Reduced levels of callose deposition were observed in the sieve elements from the infected transgenic midribs compared to the WT controls (Figure 5a). Statistical analysis showed that in the healthy plants, most of the transgenic lines had lower levels of callose compared to the WT controls (Figure 5b). After Las infection, callose deposition increased in all plants was tested but the levels in transgenic plants were shown to be significantly lower than in WT plants. The OE13 and OE28 lines displaying the strongest HLB tolerance (Figure 3) had the lowest levels of callose accumulation (Figure 5b).

### 2.5. CsSAMT1 Overexpression Enhances the Expression of Disease Resistance Genes in Citrus

To understand the molecular mechanisms underlying HLB tolerance in transgenic plants overexpressing *CsSAMT1*, the transcriptional profiles of the OE13 and OE28 lines that showed high levels of tolerance were compared to WT controls using RNA-seq (Supplementary Data S1 and Tables S2 and S3). In total, 2127 differentially expressed genes (DEGs) were identified in the OE13 line and 2429 DEGs were identified in the OE28 line (Table S4). Of these genes, 1417 and 1517 DEGs were upregulated by *CsSAMT1* overexpression in the two lines, respectively (Table S4). To validate the RNA-seq results, the expression of 34 randomly selected DEGs was investigated by RT-qPCR (Table S5). The data showed the expression patterns of these DEGs were similar to those observed in the RNA-seq data (Figure S6).

**Figure 6:**
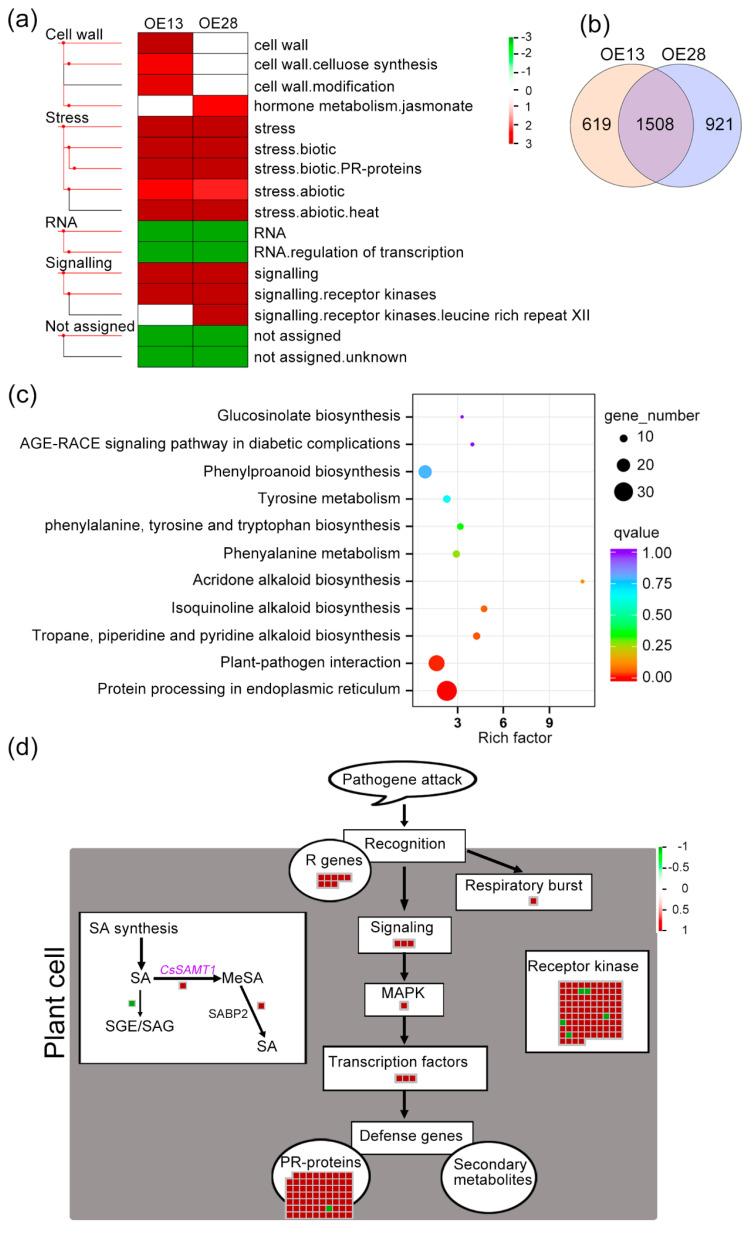
The global transcription profiles affected by *CsSAMT1* overexpression in the OE13 and OE28 lines. (**a**) PageMan comparison of differentially represented pathways and functional categories between the differentially expressed genes (DEGs) in the OE13 and OE28 lines. Rectangular blocks denote the MapMan pathway or functional categories. Up- and downregulated categories are shown in red and green, respectively. The categories differentially represented in the transgenic plants are indicated on the right. (**b**) Venn analysis of DEGs from the OE13 and OE28 lines. 1508 DEGs were shared by the two lines. (**c**) KEGG enrichment of the 1508 DEGs shared by the OE13 and OE28 lines indicating the 11 representative KEGG pathways. (**d**) MapMan visualization of DEGs involved in the defense response of transgenic citrus plants. The 1508 DEGs were visualized using the MapMan tool. Every square block indicates a DEG and significantly up- and downregulated DEGs are displayed in red and green, respectively. SA, salicylic acid; MeSA, methyl salicylate; SABP2, salicylic acid-binding protein 2; SGE, SA glucose ester; SAG, SA glucoside. The scale bar represents log2 fold change values.

MapMan analysis showed that “Cell wall”, “Stress”, “RNA”, “Signaling”, “Not assigned” pathway or function categories were significantly affected by *CsSAMT1* overexpression (Figure 6a and Supplementary Data S2). In both the OE13 and OE28 lines, “stress.biotic”, “stress.biotic.PR-proteins”, “stress.abiotic”, and “stress.abiotic.heat” pathways or functions in the stress category and “signaling.receptor kinases” in the signaling category were positively regulated by *CsSAMT1* overexpression. Also, “RNA.regulation of transcription” in the RNA category was negatively regulated by *CsSAMT1* overexpression (Figure 6a and Supplementary Data S2). KEGG enrichment further confirmed that the “plant–pathogen interaction” pathway was positively regulated by *CsSAMT1* overexpression in both lines (Figure S7 and Supplementary Data S3). Venn analysis showed that 1508 DEGs had similar expression profiles in the two lines (Figure 6b and Supplementary Data S3). Among these genes, 32 genes were significantly represented in the “protein processing in endoplasmic reticulum” process and 25 genes were significantly represented in the “plant-pathogen interaction” process (Figure 6c and Supplementary Data S3). GO enrichment also showed that the “defense response” biological process was significantly upregulated by the overexpression of *CsSAMT1* (Figure S8 and Supplementary Data S4). These data indicated that *CsSAMT1* overexpression positively regulates the plant defense response in citrus.

We then analyzed how these DEGs act in the citrus defense response using the MapMan tool (Figure 6d and Supplementary Data S5). We detected a DEG (Cs7g29470) encoding a SABP2 enzyme that transfers MeSA to bioactive SA to promote SAR defense [19]. Its expression was upregulated by *CsSAMT1* overexpression. We also found that the expression of a DEG (Cs2g18240) that encodes a UDP-glucosyl transferase (named as CsUGT74F1 in this study) was downregulated by *CsSAMT1* overexpression. UGT74F1 inactivates SA by mediating esterification or glycosylation of SA [30]. The expression profiles of the two DEDs in the OE13 and OE28 lines were further confirmed by RT-qPCR (Figure S6). In the defense response process, all DEGs (except for a PR-proteins gene) were upregulated by *CsSAMT1* overexpression. These genes were distributed into the whole defense response process as follows: pathogen recognition, respiratory burst, signaling, MAPK-mediated signal amplification, regulation of gene transcription, and finally activation of defense genes (Figure 6d). Also, most of the receptor kinase genes related to the defense response were upregulated in the transgenic lines.

Gene function annotation (Supplementary Data S5) identified eight annotated “R genes” (involved in pathogen recognition) and three transcription factors belonging to the TIR-NBS-LRR class of disease resistance genes that play roles in the plant innate immune system [31]. In signaling pathways, two DEGs were involved in jasmonate signaling (Supplementary Data S5). A serine/threonine-protein kinase *bri1* homology (cs2g30810) was assigned to the MAPK-mediated signal amplification. Most of the activated “PR-proteins” genes were also TIR-NBS-LRR members. No secondary metabolite-related genes participating in the defense response were detected in the survey. Visualization analysis clearly showed that overexpression of *CsSAMT1* enhanced the transcriptional activities of disease resistance genes in Wanjincheng oranges.

## 3. Discussion

In this study, we characterized *CsAMT1* that encodes a key enzyme known to produce the SAR signal MeSA in response to Las infection. We showed that CsAMT1 has a specific enzymatic activity for the conversion of SA to MeSA and that Wanjincheng oranges overexpressing *CsSAMT1* had increased levels of SA and MeSA. Our results confirmed that the overexpression of *CsSAMT1* enhanced the tolerance of Wanjincheng oranges to HLB. Transgenic plants had a significantly reduced Las titer and displayed alleviated chlorosis symptoms during 24 months of Las inoculation in the greenhouse. Phloemic cell over-proliferation, starch over-accumulation and callose over-deposition were found to be significantly reduced in transgenic plants compared to WT controls after Las infection. Based on RNA-seq data of the transgenic plants, our study suggested that the enhanced signaling between SA and MeSA mediated by *CsSAMT1* overexpression elevated the transcription activities of disease resistance genes that act to enhance citrus HLB tolerance.

### 3.1. CsSAMT1 Is a Specific Enzyme Converting SA to MeSA

The SABATH methyltransferase family includes O- and N-methyltransferases [32]. CsSAMT1 belongs to the O-methyltransferase family that includes SAMT, JMT, and IAMT [21]. Alignment of the amino acid sequences revealed that the SA, JA and IAA binding residues in CsSAMT1, AtJMT and AtIAMT were highly conserved. We then used an in vitro methylation assay to determine CsSAMT1 enzymatic specificity. Our results showed that CsSAMT1 is a specific enzyme converting SA to MeSA. Specific residue substitutions including Tyr-147 (Y) to Ser (S) and Met-150 (M) to His (H) at the seven hormone-binding residues site (SSYSLMW) (Figure 1a) in Clarkia SAMT confer significant methylation activity against JA. This activity is further enhanced by the residue substitutions of Ile-225 to Gln (Q) and Phe-347 to Y [21]. The quadruple mutant markedly reduces methylation activity against SA [21]. At the “SSYSLQW” site of CsSAMT1 (Figure 1a), the M residue is replaced with a Gln (Q), but this change did not affect enzymatic specificity.

In the active-site cavity of AtIAMT, the most striking change was that Trp (W) was replaced by a Gly (G) residue at position 226 relative to Clarkia SAMT (Figure 1a). This substitution creates a large and spacious pocket for the recognition and binding of the indole ring of IAA [21] indicating that the three-dimensional structure of the substrate-binding pocket is a strong determinant of enzymatic specificity of the SABATH family. Further studies are required to fully elucidate how these key residues affect the activity and specificity of CsSAMT1.

### 3.2. Roles of SA and MeSA in Response to HLB Disease in Citrus

Several studies have indicated that SA-mediated innate defenses play central roles in citrus response to HLB [10,11,17,33]. Previously, we showedthat HLB-tolerant Sour pomelo has high levels of SA and MeSA compared to HLB-susceptible Jincheng oranges. We have also shown that the SA contents dropped dramatically in Sour pomelo and conversely increased in Jincheng oranges after Las infection [13]. The presented study showed that overexpression of *CsSAMT1* significantly increased both MeSA and SA contents in transgenic citrus plants and synchronously enhanced plant tolerances to HLB. Similarly, in tomatoes, *SAMT* overexpression elevates both SA and MeSA accumulation and promotes plant disease resistance [24]. However, in soybean and *Arabidopsis*, *SAMT* overexpression causes an increase in MeSA but a drop in SA levels that leads to a weakening of disease resistance in *Arabidopsis* but enhances resistance in soybean [22,23,26]. These results showed that *SAMT1* has different roles in the regulation of MeSA and SA -mediated disease resistance in different species or varieties. It has also been shown that the exogenous application of MeSA leads to plant disease resistance [34,35]. The defense function of MeSA is mainly due to the increase or alteration of endogenous SA levels [19,35]. Based on the above data, we suggest that high basal levels of SA and MeSA are favorable for enhanced citrus tolerance to HLB.

### 3.3. Effects of CsSAMT1 Overexpression on SA Metabolism in Citrus

In plants, MeSA is synthesized only from SA [36] and so the MeSA overproduction induced by CsSAMT1 may consume SA. These changes in turn trigger SA accumulation as shown in transgenic citrus plants overexpressing *CsSAMT1* (Figure 2g). Plant SA biosynthesis occurs via two distinct branches. In one of these routes, known as the cinnamic acid pathway, phenylalanine ammonia-lyase (PAL) is responsible for the conversion of phenylalanine to cinnamic acid. The intermediate then undergoes hydroxylation or oxidation to ortho-coumaric acid or benzoic acid which are finally oxidized or hydroxylated into SA [37]. Our RNA-seq data did not detect expression changes of the *PAL* homologies. The majority of pathogen-induced SA production occurs via isochorismate production in which Isochorismate synthase (ICS) is the critical key enzyme. In soybean, the expression of *GmICS1* and *GmICS2* was induced by overexpressing *GmSAMT1* [22]. However, no *ICS* genes were differentially expressed in our RNA-seq datasets. From our RNA-seq and RT-qPCR data, we showed that *CsUGT74F1*, which is known to inactivate SA [30,38], was downregulated by *CsSAMT1* overexpression. These data indicated that overexpression of *CsSAMT1* represses esterification or glycosylation of SA which is beneficial to the accumulation of free SA. The overexpression of the UGT74F1 homologous gene *AtSGT1* in *Arabidopsis* reduced SA accumulation and increased plant susceptibility to *Pseudomonas syringae* [39]. In tobacco, overexpression of *SAGT* also reduced SA levels and increased plant susceptibility to the cucumber mosaic virus strain Y whilst RNAi-mediated silencing of *SAGT* enhanced resistance [40]. These results suggest that the repression of *CsUGT74F1* expression in *CsSAMT1* overexpressing transgenic plants enhances tolerance to HLB by elevating SA levels. However, *OsSGT1* silencing in rice does not increase resistance to blast disease but significantly reduces the probenazole-dependent development of resistance [41]. Moreover, our study showed that *CsSAMT1* overexpression can enhance the expression of *SABP2* (Cs7g29470). SABP2 is responsible for conversion of MeSA to SA and this accumulation of SA activates SAR response in plant [19,20]. The data indicated that *CsSAMT1* overexpression can enhance SA accumulation through activating SABP2-mediated conversion of MeSA to SA, which is beneficial to citrus tolerance to HLB disease. The contribution of *CsUGT74F1* and *SABP2* to the HLB resistance of citrus plants remains to be fully determined.

### 3.4. CsSAMT1 Overexpression Enhanced Defense Response through Upregulating Transcription of Disease Resistance Genes

The innate immune system in plants consists of a series of receptor proteins that monitor extra- and intracellular pathogen-related signals to activate plant defenses. The receptor-like kinase (RLK) and receptor-like proteins (RLPs) recognize apoplastic signals from pathogens and host damage that are transduced across the plasma membrane to trigger PTI-mediated defenses [42]. In plant cells, intracellular receptors recognize virulent proteins (such as type III effector proteins) delivered by pathogens to activate ETI-mediated defense [43]. These intracellular receptors are NBS-LRR proteins that are characterized by nucleotide-binding site (NBS) domains and a C-terminal Leucine-rich repeat (LRR) [44].

Most of the disease resistance genes in plants identified to date encode NBS-LRR proteins [44]. Our transcriptomic analysis showed that *CsSAMT1* overexpression led to significantly increased expressions of several RLK/RLPs and NBS-LRR genes (Supplementary Data S5). These data indicated that both PTI- and ETI-mediated resistance were enhanced in the transgenic plants. There are two known subfamilies of plant NBS-LRR proteins defined by the presence of Toll/interleukin-1 receptor (TIR) or coiled-coil (CC) motifs in the amino-terminal domain [7,44]. Gene function annotation showed that most of the NBS-LRR genes activated by *CsAMT1* overexpression were TIR-NBS-LRR members. No pathogenesis or secondary metabolites relating to defense genes were induced by overexpression of *CsSAMT1*.

TIR-NBS-LRR and CC-NBS-LRR proteins usually function through distinct signaling pathways. TIR-NBS-LRRs regulate the defense response through the ‘Enhanced Disease Susceptibility’ protein EDS1 whilst CC-NBS-LRRs act through the ‘Non-race specific Disease Resistance’ protein NDR1 [7]. The Arabidopsis CC-NBS-LRRs RPP8 and RPP13 also activate a separate defense pathway independent of EDS1 and NDR [45]. EDS1 is critical for disease resistance in *Arabidopsis* and other plants and plays a role in SA-mediated defense response [46,47,48]. Zheng and Zhao [49] showed that many HLB responsive genes including *NBS-LRR*s connect to EDS1 and EDS1-mediated defense in citrus. In our RNA-seq data, a *RPS4* homology (Cs5g22500) that belongs to the TIR-NBS-LRR family showed increased expression (Supplementary Data S5). In *Arabidopsis*, RPS4 is associated with EDS1 which forms an immune receptor complex against the AvRps4 effector of *p. syringae* [50,51]. Another TIR-NBS-LRR gene *RPS2* (Cs3g06500) and a CC-NBS-LRR gene *RPM1* (Cs4g08020) were also upregulated by *CsSAMT1* overexpression (Supplementary Data S5). *RPS2* and *RPM1* homologies in *Arabidopsis* confer resistance responses against *p. syringaes* expressing the effector genes *avrRpt2* [52] and *avrRpm1* or *avrB* [53], respectively. Collectively, our data showed that NBS-LRRs, particularly the TIR-NBS-LRR class, have a potentially important role in the CsSAMT1-mediated defense response to HLB in citrus.

In summary, we provide clear evidence that overexpression of *CsSAMT1* positively regulates citrus HLB resistance by enhancing levels between SA and MeSA and subsequently activating the expression of disease resistance genes (especially, *TIR-NBS-LRRs*). Our data provide insight for improving citrus resistance to HLB by manipulation of SA in citrus breeding. We highlight that enhanced tolerance to Las in transgenic plants was confirmed by the grafting method in a greenhouse. Also, HLB disease is spread naturally by ACPs [1]. Further investigations are underway to determine whether the HLB tolerance of transgenic plants can be sustained in the field by exposure to free-flying Las-positive ACPs and if this strategy is a commercially viable approach.

## 4. Materials and Methods

### 4.1. Plant and Las Bacteria Materials and Growth Conditions

Wanjincheng oranges (*C. sinensis* Osbeck) and all transgenic plants used in this study were planted in a greenhouse with a 16 h photoperiod of 45 µmol m^−2^s^−1^ illumination with 60% RH at the National Citrus Germplasm Repository, Chongqing, China. Wanjincheng oranges containing *Candidatus* Liberibacter asiaticus (Las) were also maintained in a greenhouse with restricted access. The 16s-f/16s-r (Table S6) primers were used to confirm the presence of the Las pathogen by PCR.

### 4.2. DNA Constructs

The coding sequence of *CsSAMT1* [13] was cloned from Wanjincheng oranges using the primers SAMT-f/SAMT-r (Table S6) and inserted into the pGEM-T Easy vector (Promega, Madison, WI, USA) to generate pGE-SAMT1. The obtained *CsSAMT1* sequence was identified by Sanger sequencing. To express the CsSAMT1 protein in yeast, *CsSAMT1* was amplified from the pGE-SAMT1 with the primers PIKSAMT-f/PIKSAMT-r (Table S6) and inserted into the pPIC9K vector to produced pK-SAMT1. To construct the plant overexpression vector containing the *CsSAMT1* gene, this gene was excised from the pGE-SAMT1 vector with *Kpn*I/*Sal*I and inserted into the plant expression vector pGN [29] to generate the p35S::SAMT1 plasmid in which *CsSAMT1* expression was driven by the strong constitutive promoter 35S. The plant overexpression vector p35S::SAMT1 was then transformed into the *Agrobacterium tumefaciens* strain EHA105 by electroporation. All the constructions were confirmed by PCR, restriction endonuclease digestion, and sequencing analysis.

### 4.3. Multiple Sequence Alignments

Multiple sequence alignment of CsSAMT1 with selected known SAMT proteins was performed using the Blast program (NCBI, Bethesda, MD, USA). The conserved domains in the amino acid sequence of CsSAMT1 were determined based on known SAMT proteins.

### 4.4. Protein Expression and Enzyme Activity Analysis

To express the CsSAMT1 protein in yeast, the pPIC9K-SAMT1 plasmid was transferred into *Pichia pastoris* GS115. Yeast transformation, colony screening, induced expression, and purification were performed according to the manufacturer’s instructions (Invitrogen, Carlsbad, CA, USA). The purity of expressed proteins was determined by SDS-PAGE and the protein concentrations were determined by the Bradford assay [54].

The activity levels of CsSAMT1 were performed by activity assays according to the protocol of Zubieta et al. [21]. A 50 μL volume was prepared containing 50 mM of Tris-HCl (pH 7.5), 1 mM SA, 2 mM of S-adenosyl-L-Met (SAM) as a methyl donor and 1 μg of CsSAMT1 protein. The assay was initiated by the addition of CsSAMT1 and maintained at 25 °C for 30 min. The reaction was stopped by the addition of ethyl acetate (200 μL). The SA and MeSA contents in the reaction mixture were determined by SA and MeSA ELISA (enzyme-linked immunosorbent assay) kits (Jiweibio, Shanghai, China). The enzyme activity of CsSAMT1 was expressed as the amount of synthesized MeSA.

The substrate specificity of recombinant CsSAMT1 against SA, JA, and IAA was determined as previously described [21]. Different amounts of substrate (0, 250, 500, 750, 1000, 1250, 1500, 1750, and 2000 µM) were added into the above reaction mixture. After 30 min of inoculation, the MeSA, methyl jasmonic acid (MeJA) and methyl indole-3-acetic acid (MeIAA) contents in the reaction mixture were determined using enzyme-linked immunosorbent assay (ELISA) kits for MeSA, MeJA and MeIAA (Jiweibio, Shanghai, China). The kinetic properties of CsSAMT against the substrate were determined by fitting the initial velocity against the substrate concentrations to the hyperbolic Michaelis-Menten equation using GraphPad Prism version 5.0 (GraphPad Software, SanDiego, CA, USA). The maximum velocities (V_max_) were converted to apparent K_cat_ values (turnover numbers) and expressed in units of s^−1^. Errors in both K_cat_ and K_m_ were calculated in GraphPad Prism. The assay was repeated three times.

### 4.5. Citrus Transformation

The epicotyls of Wanjincheng oranges were used as explants for citrus transformation. The transformation protocol and identification of transgenic plants were performed according to the protocol of Zou et al. [29]. The transgenic plants were recovered by grafting onto Troyer citrange (*Poncirus trifoliata* (L.) Raf. × *C. sinensis* Osbeck) seedlings in vitro. All transgenic and WT plants were further grafted onto two-year old Troyer citrange seedlings and grown in a netted greenhouse with a 16 h photoperiod of 45 µmol m^−2^s^−1^ illumination with 60% RH at 28 °C.

### 4.6. qRT-qPCR Analysis

Total RNA from the citrus samples was isolated using the EASYspin Plant RNA Extraction Kit following the manufacturer’s instructions (Aidlab, Beijing, China). The RNA from the leaf tissues was used to determine the expression levels of *CsSAMT1* in the transgenic lines. RNA was reverse transcribed into the first cDNA using the iScriptTM cDNA Synthesis Kit (Bio-Rad, Hercules, CA, USA). Gene expression was detected using the iQ™ SYBR Green Supermix (Bio-Rad). The PCR reactions were carried out by a pretreatment (94 °C for 5 min) followed by 40 amplification cycles (94 °C for 20 s and 60 °C for 60 s). The primers used in the qRT-PCR analysis are listed in Table S1 and all experiments were performed in triplicate. The expression of the citrus *actin* (GenBank No. XM_006464503.3) [13,55] and *GAPDH* [56] genes were used for transcript normalization. Using WT plants as controls, the relative expression of *CsSAMT1* in transgenic plants was calculated by the 2^−ΔΔCt^ method [57].

### 4.7. Measurement of Hormone and CsSAMT Protein Content in Citrus

First, 0.5 g of fresh tissues from fully mature leaves were powdered in 1.5 mL 0.9% NaCl solution and centrifuged at 3000 rpm for 10 min. Then, the supernatants were transferred into a tube and the hormone levels were detected. The levels of SA and MeSA in the supernatant were determined using the plant SA and MeSA ELISA kits (Jiweibio), respectively. CsSAMT protein was also determined using a plant methyl salicylate synthase (SAMT) ELISA detection kit (Jiweibio). The tests were performed in triplicate.

### 4.8. Evaluation of HLB Tolerance in Citrus

The evaluation of tolerance to Las in citrus plants was performed as previously described by Zou et al. [29]. To evaluate Las tolerances in the transgenic plants, transgenic and WT lines were propagated by grafting on Troyer citrange rootstock in the greenhouse. After one year, three or four plants per transgenic line including WT plants were grafted with Las-infected axillary buds. For each plant tested, 3 or 4 buds were grafted on the stem. All the inoculated plants were maintained in a greenhouse and the incidence of disease was regularly observed.

Every 3 months, three leaves per plant were selected randomly and their midrib tissues were pooled, and the DNA was isolated from the pooled tissues. The content of the Las *16S* gene in the isolated DNA samples was detected by quantitative PCR (qPCR) using the citrus *18S* gene as the internal reference [29]. The 16S-f/16S-r and 18S-f/18S-r primers (Table S6) were used to amplify the *16S* and *18S* genes, respectively. The qPCR was performed in a final volume of 10 µL, containing 5 µL 2 × iQ™ SYBR Green Supermix (Bio-Rad), 0.5 µL of each primer (10 mM) and 20 ng DNA. The PCR protocol was carried out by a pretreatment (95 °C for 2 min) followed by 40 amplification cycles (94 °C for 10 s and 60 °C for 60 s). The Las bacterial populations (Las cells μg^−1^ of citrus DNA) were determined based on the formula previously used [29]. The disease intensity of the individual transgenic lines was evaluated based on the bacterial populations in three plants.

### 4.9. Microscopic Observation and Callose and Starch Grain Analysis

Microscopic observations of the phloem structure, callose, and starch grain determinations were performed as previously described [13]. For the observations, 1 cm midrib sections of similar age, position, and developmental stage were obtained from the transgenic and WT leaves. The samples were embedded in resin 20 μm thick transverse slides made. Starch grains were detected by counterstaining with methylene blue-azure A and basic fuchsin staining. Callose was stained using 0.05% aniline blue. Callose and starch grains were observed under UV illumination and white light, respectively. Callose deposition was quantified by counting the number of fluorescent spots in the phloem of each sample [58]. Starch isolation and content determination were performed according to the protocol of the Starch Assay Kit (G-clone Biotechnology Co., Ltd.; Beijing, China). Finally, 0.1 g of fresh leaf tissue was used to quantify the starch content in the plants. All analyses were performed in triplicate.

### 4.10. RNA-seq Analysis

For RNA-Seq analysis, fully mature leaves were sampled from two transgenic lines (OE13 and OE28) and WT controls. Three biological replicates were used for each sample. Total RNA isolation, quality assessment and construction of the sequencing libraries were performed as described [59]. RNA-Seq and basic bioinformatics analyses were performed by BioMarker Technologies Illumina, Inc. (Shanghai, China). All the clean reads were mapped to the reference genome of sweet oranges (http://citrus.hzau.edu.cn/orange/index.php, accessed on 30 December 2020) using the HISAT 2.0.5 software [60]. Compared to the WT controls, differentially expressed genes (DEGs) were screened using the DESeq2 package [61]. The DEGs were defined based on a threshold of changes ≥1.5 with adjusted *p*-values < 0.05. GO function and KEGG pathway enrichment analysis of the DEGs were performed using the GOseq R package [62] and KOBAS software [63], respectively.

The MapMan tool [64] was also used to analyze citrus gene expression data using our constructed citrus MapMan mapping database [59]. Differentially represented MapMan pathways were defined using a two-tailed Wilcoxon rank sum-test and corrected using the Benjamin–Hochberg method (false discovery rate < 0.05).

## Data Availability

All RNA sequencing files are available from the SRA database (accession number PRJNA692096, https://www.ncbi.nlm.nih.gov/sra/PRJNA692096, accessed on 30 December 2020).

## Supplementary Materials

Supplementary materials are available online at https://www.mdpi.com/1422-0067/22/6/2803/s1.
