# Supplementary material for: Overexpression of Salicylic Acid Carboxyl Methyltransferase (CsSAMT1) Enhances Tolerance to Huanglongbing Disease in Wanjincheng Orange (Citrus sinensis (L.) Osbeck)
Source: Int J Mol Sci. 2021 Mar 10;22(6):2803. doi: 10.3390/ijms22062803 (PMC7999837; doi:10.3390/ijms22062803)
Supplement: Supplementary file 1 [file ijms-22-02803-s001.zip › ijms-1113839-SL/Supplementary Files/Supplementary Materials.docx]

**Overexpression of salicylic acid carboxyl methyltransferase (*CsSAMT1)* enhances tolerance to Huanglongbing disease in citrus**

Xiuping Zou^1,†^, Ke Zhao^1^, Yunuo Liu^1^, Meixia Du^1^, Lin Zheng^1^, Shuai Wang^1^, Lanzhen Xu^1^, Aihong Peng^1^, Yongrui He^1^, Qin Long^1^, Shanchun Chen^1,†^

^1^ Citrus Research Institute, Southwest University/Chinese Academy of Agricultural Sciences, Chongqing, P. R.China

**Table S1** Quantification of the Las populations in the transgenic lines by qPCR

|  | Ct value of Las 16S gene | | |
| --- | --- | --- | --- |
| Line | 12 MAI | 18 MAI | 24 MAI |
| WT | 20.22±1.17^a^ | 17.31±1.92^a^ | 16.84±0.17^a^ |
| OE1 | 20.84±0.1^a^ | 17.85±0.78^a^ | 19.23±0.4^ab^ |
| OE13 | 31.28±0.06^c^ | 32.44±1.23^c^ | 28.97±2.16^cd^ |
| OE23 | 31.10±0.43^c^ | 30.44±2.94^bc^ | 26.19±2.4^bcd^ |
| OE24 | 26.83±1.28^b^ | 24.41±1.38^ab^ | 22.70±0.57^abc^ |
| OE28 | 28.20±1.89^b^ | 33.65±0.6^c^ | 33.38±0.7^6d^ |
| OE31 | 31.58±0.35^c^ | 26.57±1.18^bc^ | 24.44±0.71^bc^ |

The mean threshold cycle values (Ct) at 12, 18, and 24 months after infection (MAI) were determined by qPCR. Standard errors were calculated from three or four plants per line. Different letters on top of the bars indicate significant differences from the wildtype (WT) control based on a Tukey’s test (P < 0.05).

**Table S2** Summary of the sequencing data obtained for each sample

| Samples | Clean reads | Clean bases | GC Content | %≥Q30 |
| --- | --- | --- | --- | --- |
| OE131 | 19,928,555 | 5,965,862,126 | 45.47% | 93.99% |
| OE132 | 21,671,615 | 6,485,386,242 | 44.27% | 94.04% |
| OE133 | 22,314,144 | 6,675,403,988 | 44.51% | 94.14% |
| OE281 | 21,863,424 | 6,544,009,250 | 45.30% | 93.73% |
| OE282 | 21,757,043 | 6,507,221,264 | 44.54% | 94.14% |
| OE283 | 22,184,843 | 6,633,755,170 | 44.44% | 93.67% |
| WT1 | 31,031,794 | 9,270,869,194 | 44.79% | 93.54% |
| WT2 | 33,434,060 | 9,989,549,752 | 44.56% | 93.53% |
| WT3 | 26,582,409 | 7,943,316,154 | 44.46% | 93.63% |

**Table S3** Mapping summary of the sequencing data for each sample

| Samples | Total Reads | Mapped Reads (%) | Uniquely Mapped Reads (%) | Multiple Mapped Reads (%) |
| --- | --- | --- | --- | --- |
| OE131 | 39,857,110 | 36,169,037 (90.75%) | 32,692,826 (82.03%) | 3,476,211 (8.72%) |
| OE132 | 43,343,230 | 39,863,545 (91.97%) | 37,415,711 (86.32%) | 2,447,834 (5.65%) |
| OE133 | 44,628,288 | 40,661,579 (91.11%) | 38,021,091 (85.20%) | 2,640,488 (5.92%) |
| OE281 | 43,726,848 | 39,705,421 (90.80%) | 35,512,193 (81.21%) | 4,193,228 (9.59%) |
| OE282 | 43,514,086 | 39,891,071 (91.67%) | 37,601,513 (86.41%) | 2,289,558 (5.26%) |
| OE283 | 44,369,686 | 40,755,115 (91.85%) | 38,431,573 (86.62%) | 2,323,542 (5.24%) |
| WT1 | 62,063,588 | 57,466,286 (92.59%) | 54,092,583 (87.16%) | 3,373,703 (5.44%) |
| WT2 | 66,868,120 | 61,933,640 (92.62%) | 58,392,537 (87.32%) | 3,541,103 (5.30%) |
| WT3 | 53,164,818 | 49,119,903 (92.39%) | 46,351,046 (87.18%) | 2,768,857 (5.21%) |

**Table S4** Statistic analysis of the differentially expressed genes (DEGs) in the OE13 and OE28 lines

| Sample | No. of DEGs | No. of upregulated gene | No. of downregulated gene |
| --- | --- | --- | --- |
| OE13_vs_WT | 2127 | 1417 | 710 |
| OE28_vs_WT | 2429 | 1517 | 912 |

**Table S5** Different expression genes (DEGs) validated by qRT-PCR

| GeneID | OE13 line | | | | | OE28 line | | |
| --- | --- | --- | --- | --- | --- | --- | --- | --- |
|  | Log2(Fold change) | | FDR | | | Log2(Fold change) | | FDR |
| Upregulated |  | |  | | |  | |  |
| Cs1g17210 | 1.62 | | 1.75E-10 | | | 2.15 | | 6.69E-47 |
| Cs2g18240 | -1.51 | | 1.99E-12 | | | -1.58 | | 3.32E-34 |
| Cs3g12370 | 1.03 | | 1.31E-03 | | | 2.00 | | 1.60E-15 |
| Cs3g13340 | 2.02 | | 1.16E-35 | | | 1.86 | | 1.55E-37 |
| Cs5g17510 | 1.71 | | 1.04E-07 | | | 2.39 | | 9.53E-175 |
| Cs5g19230 | 2.18 | | 1.88E-14 | | | 1.62 | | 4.61E-23 |
| Cs5g19370 | 1.67 | | 9.40E-11 | | | 2.71 | | 3.45E-42 |
| Cs5g19460 | 1.98 | | 6.83E-21 | | | 2.22 | | 1.46E-37 |
| Cs6g15650 | 2.86 | | 1.22E-12 | | | 1.81 | | 1.57E-05 |
| Cs7g21370 | 1.91 | | 1.82E-05 | | | 2.16 | | 1.46E-10 |
| Cs7g30720 | 2.07 | | 4.23E-19 | | | 1.85 | | 9.08E-82 |
| Cs7g29470 | | 1.21 | | 7.53E-05 | 1.37 | | 2.78E-08 | |
| Cs8g12410 | 1.61 | | 1.28E-11 | | | 2.01 | | 2.19E-52 |
| Cs8g14810 | 2.36 | | 2.64E-11 | | | 2.32 | | 1.65E-30 |
| orange1.1t03893 | 2.02 | | 8.31E-20 | | | 1.48 | | 4.73E-17 |
| orange1.1t03970 | 1.34 | | 2.75E-03 | | | 2.35 | | 5.59E-12 |
| orange1.1t04673 | 1.29 | | 3.91E-03 | | | 2.03 | | 2.38E-08 |
| orange1.1t05252 | 1.97 | | 6.64E-07 | | | 2.61 | | 1.04E-17 |
| Downregulated |  | |  | | |  | |  |
| Cs1g06760 | -2.18 | | 7.48E-22 | | | -1.47 | | 1.90E-63 |
| Cs2g12490 | -2.00 | | 1.02E-07 | | | -1.87 | | 2.78E-119 |
| Cs2g13230 | -2.54 | | 3.98E-30 | | | -2.85 | | 4.92E-78 |
| Cs2g19010 | -2.52 | | 3.60E-94 | | | -2.56 | | 6.97E-147 |
| Cs3g24930 | -2.21 | | 2.17E-08 | | | -2.20 | | 1.56E-11 |
| Cs4g02210 | -1.66 | | 3.95E-03 | | | -2.19 | | 1.31E-06 |
| Cs4g13690 | -2.72 | | 5.04E-20 | | | -2.80 | | 1.69E-35 |
| Cs4g15820 | -2.84 | | 6.74E-71 | | | -3.26 | | 2.34E-167 |
| Cs6g16000 | -2.01 | | 2.98E-61 | | | -1.61 | | 4.76E-56 |
| Cs6g18930 | -2.38 | | 1.76E-06 | | | -2.79 | | 3.31E-15 |
| Cs7g02880 | -2.45 | | 3.61E-100 | | | -2.44 | | 5.35E-74 |
| Cs7g22090 | -1.18 | | 7.38E-06 | | | -2.03 | | 9.98E-15 |
| Cs8g18860 | -3.98 | | 7.20E-176 | | | -4.24 | | 8.13E-166 |
| Cs9g12370 | -3.07 | | 2.14E-83 | | | -2.94 | | 1.61E-160 |
| orange1.1t01728 | -2.43 | | 2.53E-06 | | | -1.12 | | 1.19E-02 |
| orange1.1t04712 | -2.44 | | 3.17E-107 | | | -2.38 | | 5.23E-140 |

**Table S6** Primers used in the study

| Primer name | Primer sequence (5′-3′) | Usage |
| --- | --- | --- |
| SAMT-f | act*GGTACC*GTGCGAGAGATGGAGGTGGTT | Construction of p35S::*SAMT1* vector |
| SAMT-r | gat*GTCGAC*TCATCCAATTTTCGTCAAGG |  |
| 35s-f | cgACACGCTTGTCTACTCCA | Confirmation of transgenic plants |
| samt-r | CCTTTGTTGGGAACGTGCTG |  |
| qSAMT-f | AGAATACTGTGCGAGAGATGGAG | Analysis of *CsSAMT1* expression |
| qSAMT-r | AGTGATCATCCAATTTTCGTCAAGG |  |
| *Actin*-f | CATCCCTCAGCACCTTCC | Analysis of *actin* expression |
| *Actin*-r | CCAACCTTAGCACTTCTCC |  |
| 16S-f | TGAGTGCTAGCTGTTGGGTG | Quantitative analysis of Las 16S gene |
| 16S-r | CTGCGCGTTGCATCGAATTA |  |
| 18S-f | AATTGTTGGTCTTCAACGAGGAA | Quantitative analysis of Las 18S gene |
| 18S-r | AAAGGGCAGGGACGTAGTCAA |  |
| PIKSAMT-f | ATC*TACGTA*ATGGAGGTGGTTCAAGTGCT | Construction of pK-CsSAMT1 vector |
| PIKSAMT-r | aat*GCGGCCGC*TCATCCAATTTTCGTCAAGG |  |
| F'-qrt-19230 | AATGCCTCGCAGCAAAGTCC | Expression analysis of cs5g19230 gene |
| R'-qrt-19230 | ACCTCCATCTGTTGCTGTCAA |  |
| F'-qrt-13340 | TGGCAAAATCTATGCGTGCAA | Expression analysis of cs3g13340 gene |
| R'-qrt-13340 | TCGGAACTGAAGCCCCTCT |  |
| F'-qrt- 05252 | GGGATTTGTGGTATGGGAGGT | Expression analysis of orange1.1t05252 gene |
| R'-qrt- 05252 | CCCAAAACTTCGAGAGTGCC |  |
| F'-qrt-19460 | TCTCGACAGCTGTGGTTTTGA | Expression analysis of cs5g19460 gene |
| R'-qrt-19460 | TACGTTTTCCAGGCTCGTCG |  |
| F'-qrt-04673 | GAGCATCAACCCCCGTACTT | Expression analysis of orange1.1t04673 gene |
| R'-qrt-04673 | ACTTCTACGAGGCCACTCAA |  |
| F'-qrt-15650 | AGTACCCTTCGCCTACTCGT | Expression analysis of cs6g15650 gene |
| R'-qrt-15650 | TCCTTCACCACTGATCGCAC |  |
| F'-qrt-c30720 | TTGCTATTCTGGGCGGAGTC | Expression analysis of cs7g30720 gene |
| R'-qrt-30720 | GAAGCTGTCCGCTCAATCCT |  |
| F'-qrt-03893 | TAACTTCACAGGTGAACTCCCAG | Expression analysis of orange1.1t03893 gene |
| R'-qrt-03893 | TCCAGCTTACGAAGATTGCCA |  |
| F'-qrt-17510 | ATGGGGAATGCAGGTCGTTT | Expression analysis of cs5g17510gene |
| R'-qrt-c17510 | CTTTGTACACGGCGCCAAAA |  |
| F'-qrt-03970 | TCAACTGACAGGACGTCTACC | Expression analysis of orange1.1t03970 gene |
| R'-qrt-03970 | TTGCCGAGACTTTCTGGGAC |  |
| F'-qrt-21370 | AACGTTGTGAAGGGCATGTG | Expression analysis of cs7g21370 gene |
| R'-qrt-21370 | CTAGCACCGCCAAAGTCAGC |  |
| F'-qrt-12410 | GAATGGGAGCAATGTGTGGTC | Expression analysis of cs8g12410 gene |
| R'-qrt-12410 | CTTGCCTGTTGTGGCACATT |  |
| F'-qrt-12370 | TGATGTGTCTTGCAGAGAAGAA | Expression analysis of cs3g12370 gene |
| R'-qrt-12370 | ATGAGTCGTCCCCGGTTCTA |  |
| F'-qrt-15820 | ACTGGAAGCAACAGAGAGGC | Expression analysis of cs4g15820 gene |
| R'-qrt-15820 | CTGCTGTGATGACGACGAGA |  |
| F'-qrt-12370 | GATGGATGGGGGAGCTGATG | Expression analysis of cs9g12370 gene |
| R'-qrt-12370 | GTGATGGTTGCTGTCCTCCA |  |
| F'-qrt-13230 | AAGGCTGATGCAGCACATCT | Expression analysis of cs2g13230 gene |
| R'-qrt-13230 | AGGAGGGTTCTGAAATGCCG |  |
| F'-qrt-13690 | AGCTAGGGAAGGGAGACTGG | Expression analysis of cs4g13690 gene |
| R'-qrt-13690 | TCACTAGCAAGCCGCAGAAA |  |
| F'-qrt-18930 | GGTTCCACAGCCTAATGGCA | Expression analysis of cs6g18930 gene |
| R'-qrt-18930 | TGTACCAAGCCACACACGTT |  |
| F'-qrt-19010 | CCATGGTGTCCTCCTTGTCC | Expression analysis of cs2g19010 gene |
| R'-qrt-19010 | GCCTTCTGACATCTGCCCTT |  |
| F'-qrt-02880 | GAACAAAGAAAGCGTGCCGT | Expression analysis of cs7g02880 gene |
| R'-qrt-02880 | GTGGTGGTCGCAGAACAAAC |  |
| F'-qrt-04712 | TGCTGCGCTAAATGTGAGGA | Expression analysis of orange1.1t04712 gene |
| R'-qrt-04712 | TAGGTGGAGTAGAGGACCGC |  |
| F'-qrt-02210 | CACAGCAAAGCCAGAAGAGC | Expression analysis of cs4g02210 gene |
| R'-qrt-02210 | TATGCTTGCCTAGTGACGCC |  |
| F'-qrt-01728 | TTGTTAGGTTGCGGGGACTC | Expression analysis of orange1.1t01728 gene |
| R'-qrt-01728 | CATCGTAAAGCTGCTGTCGC |  |
| F'-qrt-06760 | CGAGTCTTGCGGGTCTTCAT | Expression analysis of cs1g06760 gene |
| R'-qrt-06760 | GCCCACCCTTCCGGAATTTA |  |
| F'-qrt-16000 | GATAACTGGCCGGAGGTAGC | Expression analysis of cs6g16000 gene |
| F'-qrt-16000 | CCACTGCCTACTGCTGACAA |  |
| F'-qrt-12490 | AACAAGAATGTGGCCAACGC | Expression analysis of cs2g12490 gene |
| R'-qrt-12490 | TGCTAGAGGGTTAGCCGAGT |  |
| F'-qrt-17210 | ACTTCCCTGCAGAAAAGGCA | Expression analysis of cs1g17210 gene |
| R'-qrt-17210 | ACGTTGGAGAGAGGCACTTG |  |
| F'-qrt-14810 | GAGAGGTCTGCTCTCCTCCA | Expression analysis of cs8g14810 gene |
| R'-qrt-14810 | TGTTGTTGCACTCAACCCCT |  |
| F'-qrt-19370 | TCATCTACCTTTATTTCGGAGGC | Expression analysis of cs5g19370 gene |
| R'-qrt-19370 | GGAGGGAGTGAACCATTCAA |  |
| F'-qrt-18860 | GCCGAAGACGATCATGCAAC | Expression analysis of cs8g18860 gene |
| R'-qrt-18860 | GGCGGTACTTTTGCAAGTGG |  |
| F'-qrt-24930 | AGGCACATTGGAAGCCTTGA | Expression analysis of cs3g24930 gene |
| R'-qrt-24930 | GGAGGAAGCTGCAGTGGATT |  |
| F'-qrt-22090 | AGCAAATAAGACTGGCGGCT | Expression analysis of cs7g22090 gene |
| R'-qrt-22090 | CAATCCGTCTGGTGGCTCTT |  |
| F'-qrt-18240 | TTGGCGCATGAAGCAACGGG | Expression analysis of cs2g18240 gene |
| R'-qrt-18240 | ATTGGGGCATCGCCACCATC |  |
| F'-qrt-29470 | GCCCGGTTCACAGGAGAGAA | Expression analysis of cs7g29470 gene |
| R'-qrt-29470 | GGCATCTTGTTGCTCTGGTT |  |

Note: The sequences of restriction enzyme sites are in italics.


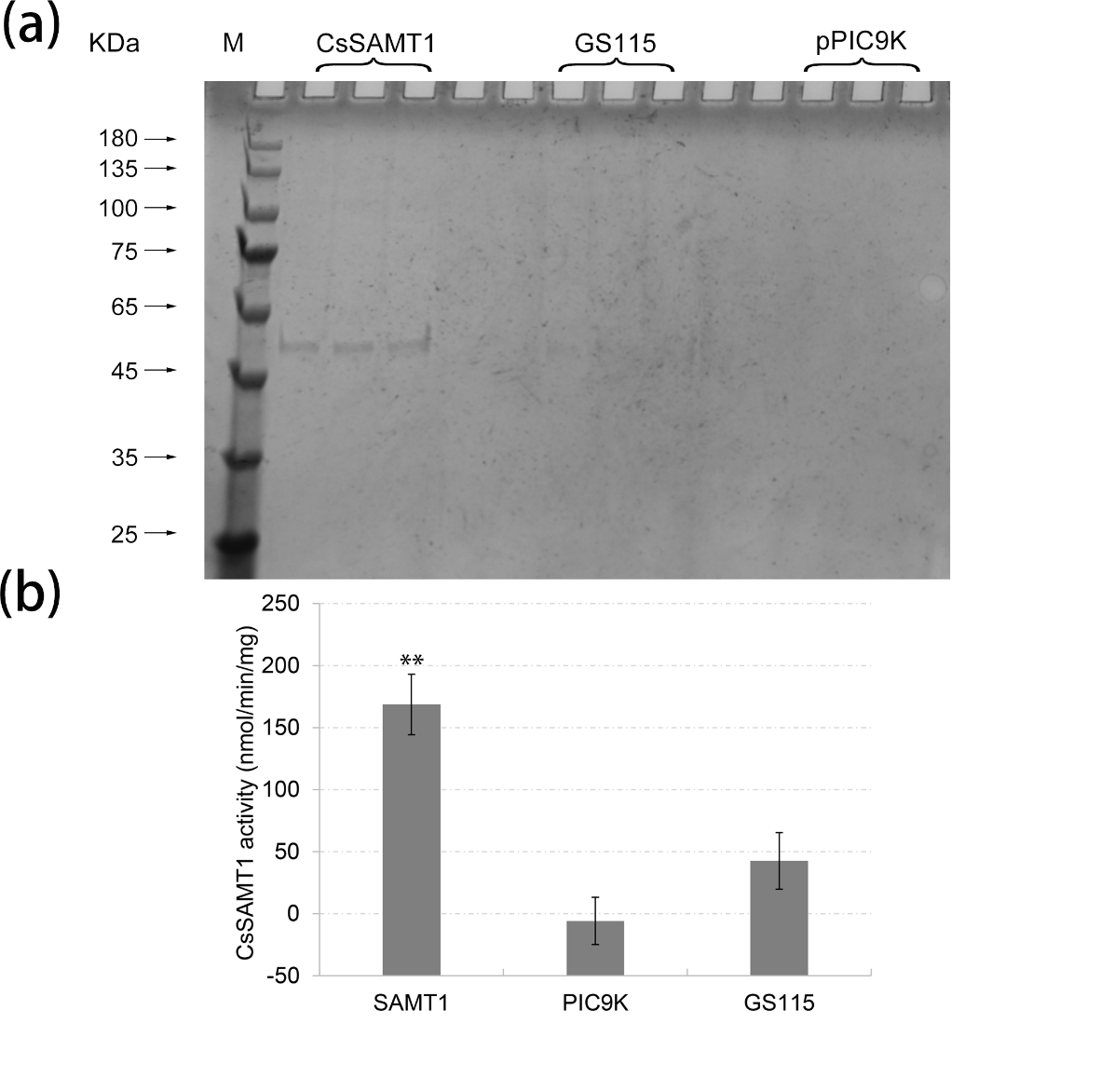


**Figure S1.** Activity of the recombinant CsSAMT1 against SA. **(a)** Purification of CsSAMT1 from the yeast GS115 (*Saccharomyces cerevisiae*) transformed with *CsSAMT1*. **(B)** Enzyme activity of recombinant CsSAMT1 with 1 mM SA as substrate and 2 mM S-adenosyl-L-Met (SAM) as methyl donor. After 30-min inoculation, synthesized MeSA contents in reaction mixture were determined using an enzyme-linked immunosorbent assay kit for MeSA (Jiweibio, China), respectively. As negative controls, the same concentrated supernatants from wildtype GS115 and GS115 transformant carrying pPICK9 empty expression vector were tested and found to contain no SAMT activity. Relative activities were calculated as a percentage of substrate with the highest activity. M, Molecular weight markers; CsSAMT1, supernatant from the *CsSAMT1*transformant; GS115, supernatant from the wildtype GS115; pPICK9, supernatant from pPICK9 control transformant. “**” showed that significant difference compared with GS115 and pPICK9 controls (T-test, *p<0.01*). The test was performed in three repeats.


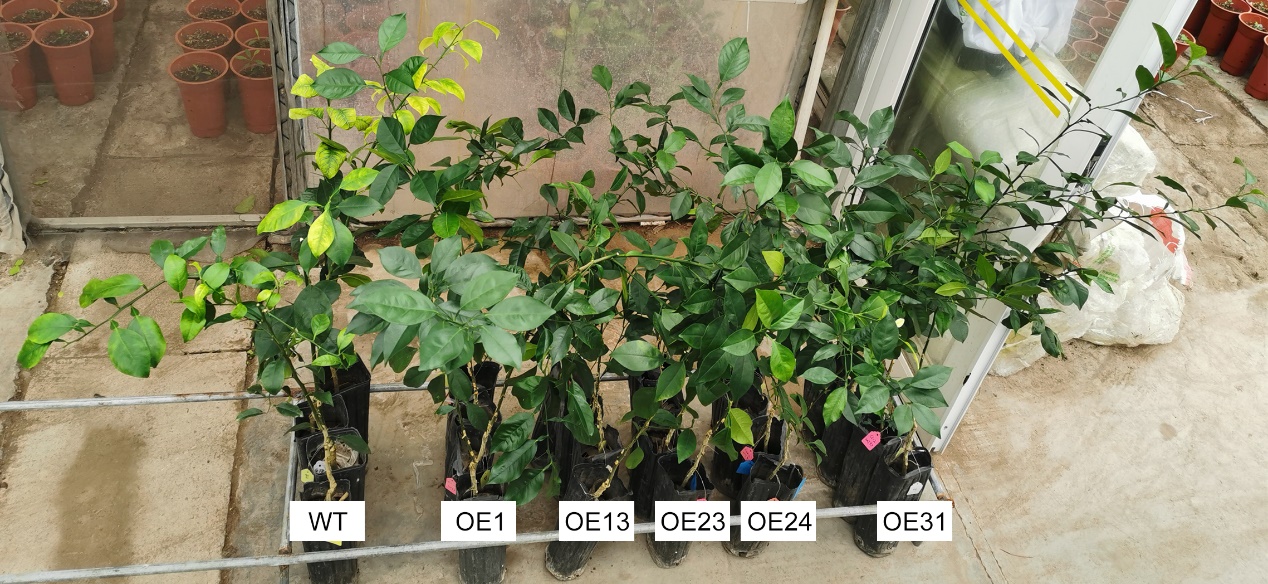


**Figure S2.** Symptoms in transgenic plants 12 months after *Candidatus* Liberibacter asiaticus infection. No obvious chlorosis was detected in transgenic lines compared with wildtype (WT) plants.


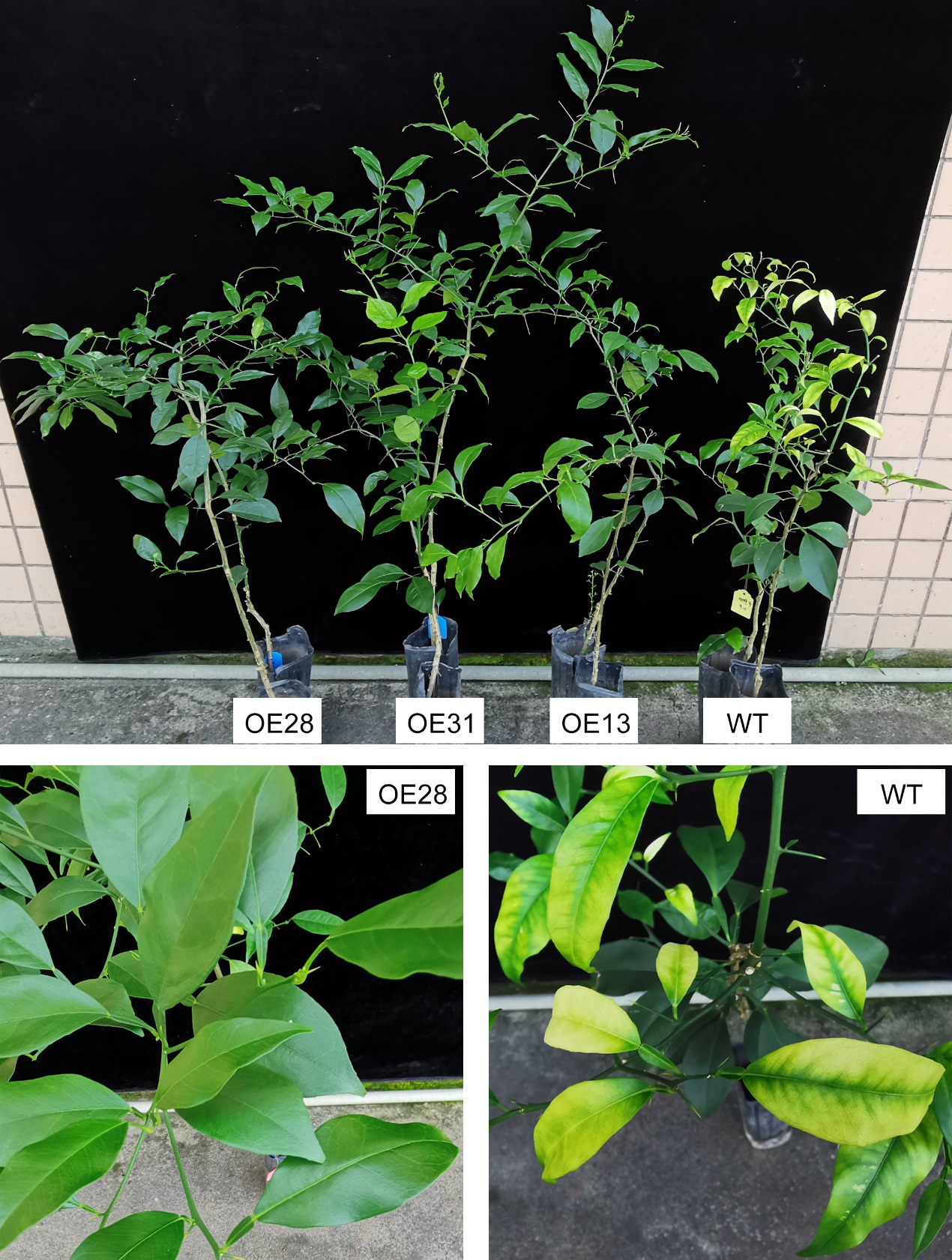


**Figure S3.** Symptoms in transgenic plants 24 months after *Candidatus* Liberibacter asiaticus infection. No obvious chlorosis was detected in OE28, OE31 and OE13 transgenic lines compared with wildtype (WT) plants.


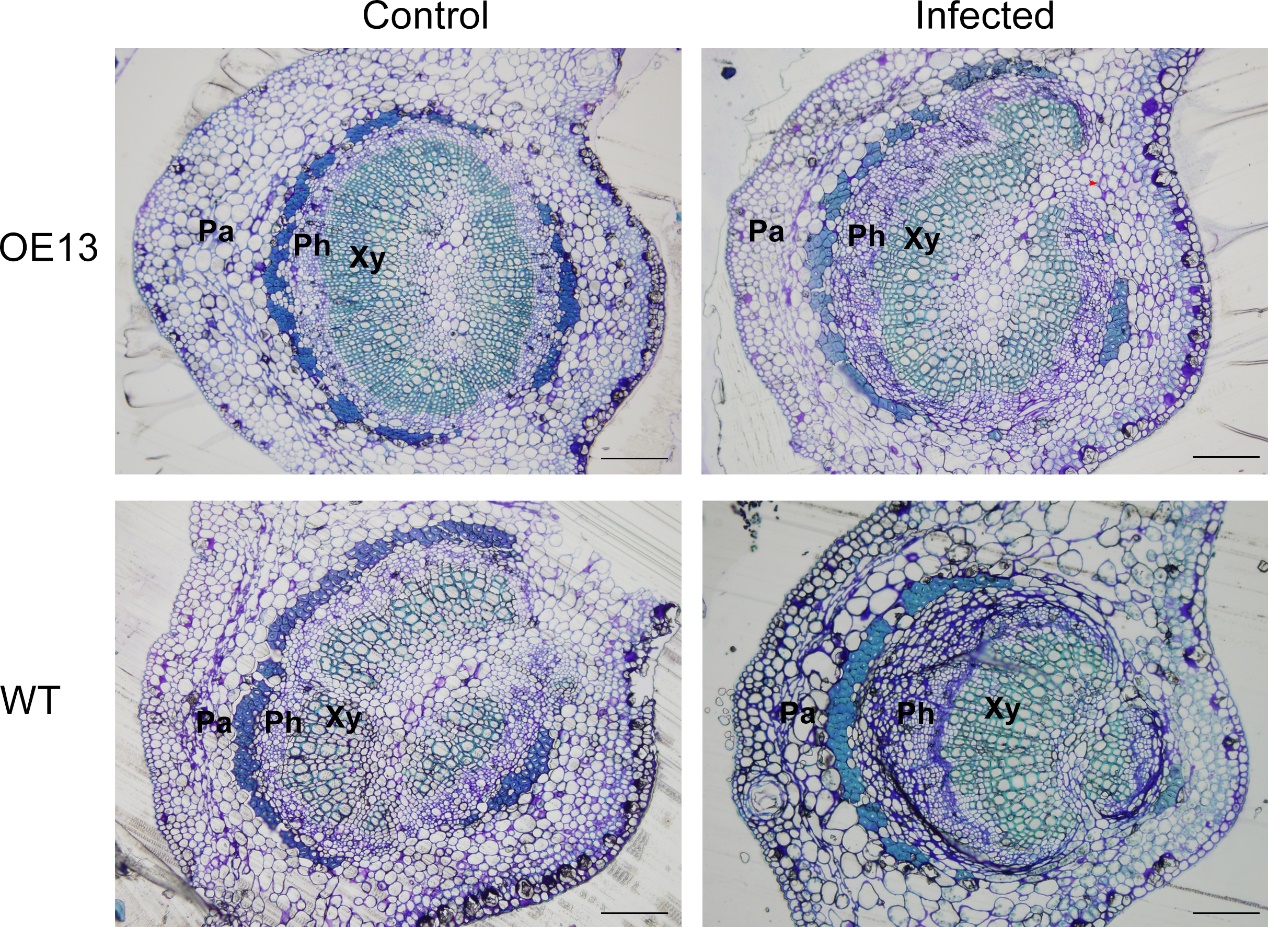


**Figure S4.** Comparison of the anatomical structures of the midrib phloem tissues between the transgenic line OE13 and wildtype plant 24 months after *Candidatus* Liberibacter asiaticus infection. The slides were stained with methylene blue-azure A and basic fuchsin. Pa, parenchyma; Ph, phloem; Xy, xylem. Bar=100 µm.


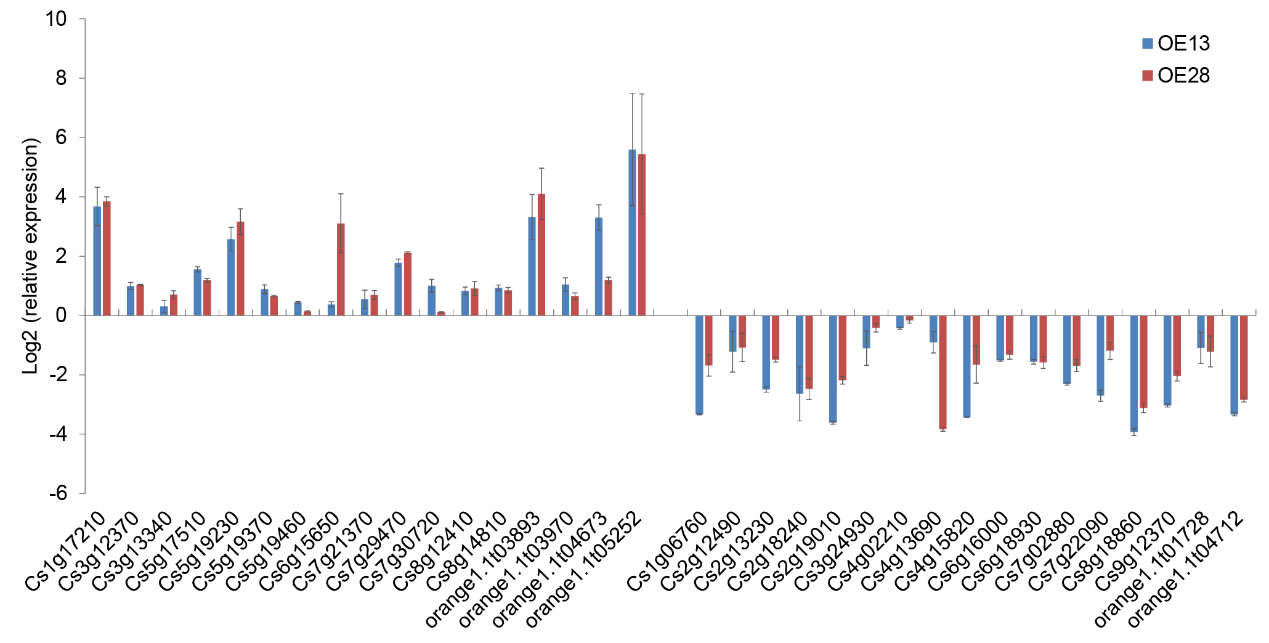


**Figure S5.** Validation of the differentially expressed genes in the OE13 and OE28 lines by RT-qPCR. Compared with wildtype control, relative expression was calculated using Actin gene as an internal control. Vertical bars indicate standard deviations of the means of three repeats.


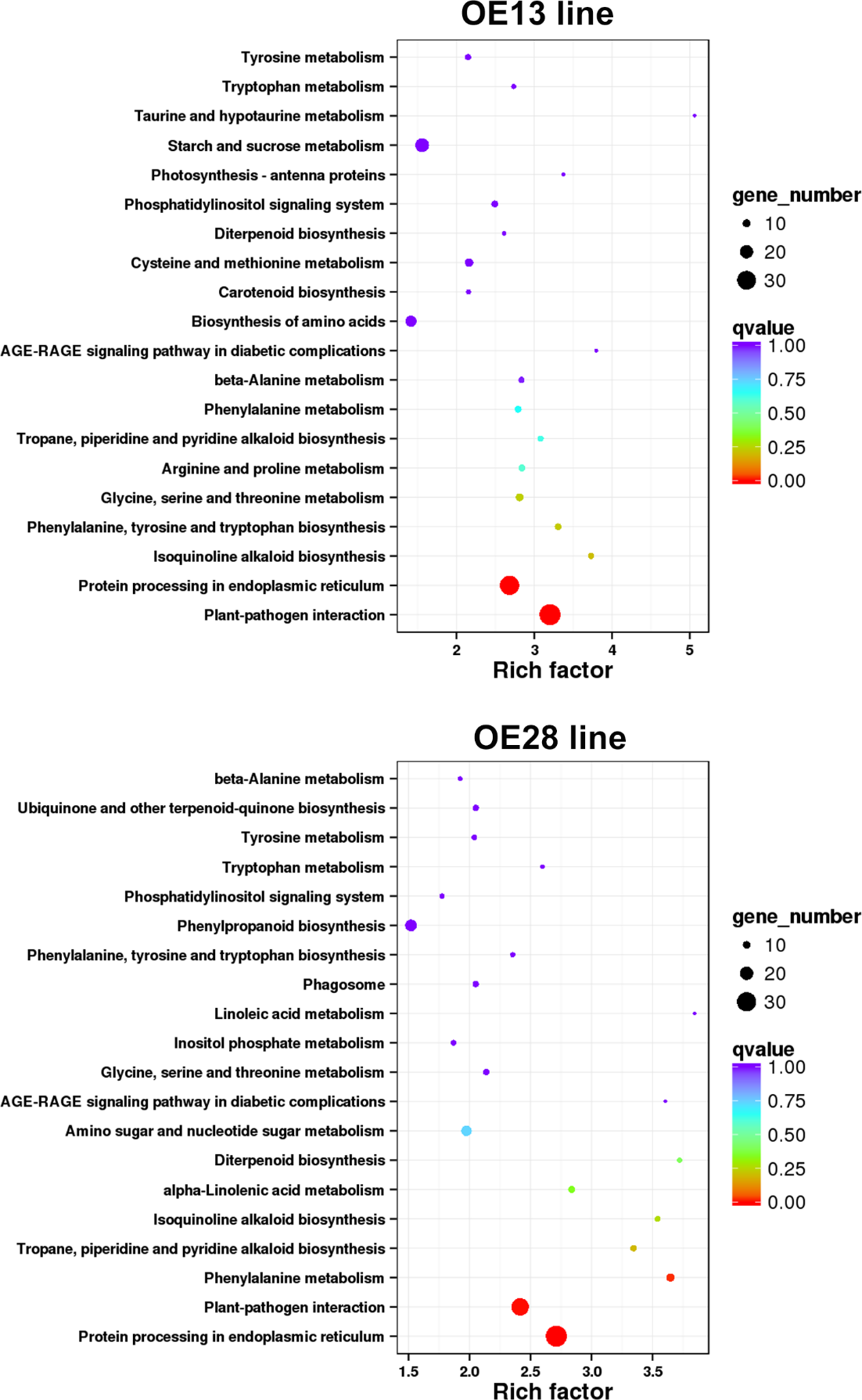


**Figure S6.** KEGG enrichment of the differentially expressed genes up-regulated by CsSAMT1 overexpression in OE13 and OE28 lines. Overexpression of *CsSAMT1* significantly upregulated genes related with “plant-pathogen interaction” and “protein processing in endoplasmic reticulum” (qvalue <0.05).


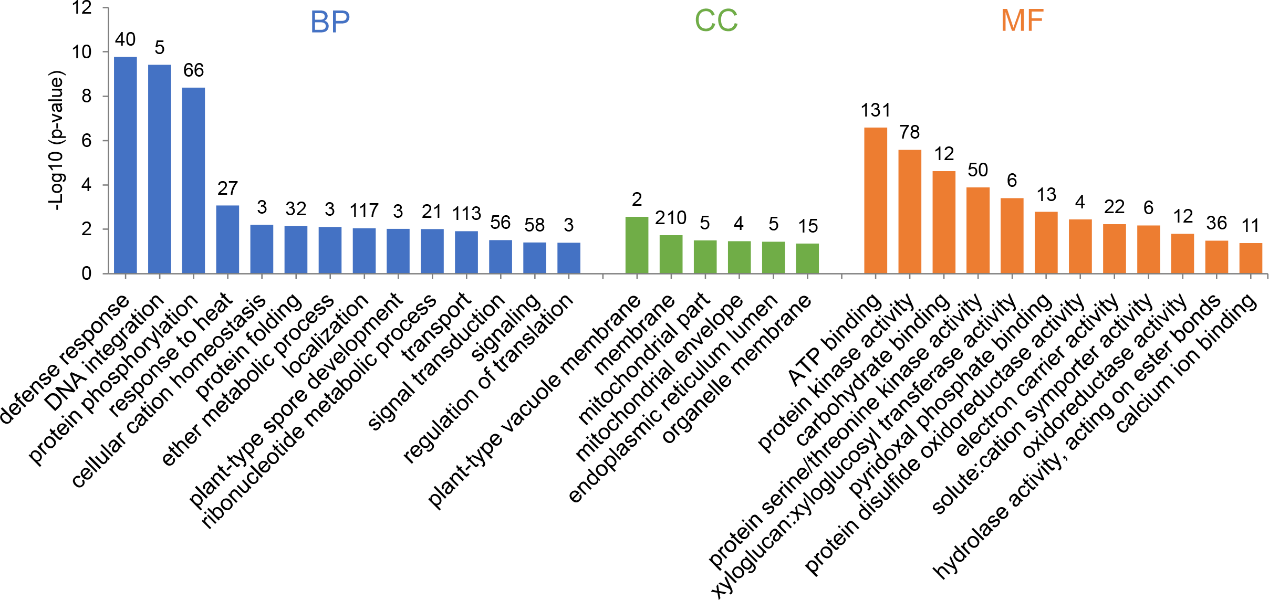


**Figure S7.** GO enrichment of the 1508 differentially expressed genes shared by the OE13 and OE28 lines. The representative GO terms were shown here.
